# Evaluation of Microwave-Assisted Extraction as a Potential Green Technology for the Isolation of Bioactive Compounds from Saffron (*Crocus sativus* L.) Floral By-Products

**DOI:** 10.3390/foods11152335

**Published:** 2022-08-05

**Authors:** Débora Cerdá-Bernad, João P. Baixinho, Naiara Fernández, María José Frutos

**Affiliations:** 1 Agro-Food Technology Department, CIAGRO-UMH, Centro de Investigación e Innovación Agroalimentaria y Agroambiental, Miguel Hernández University, 03312 Orihuela, Spain; 2 iBET, Instituto de Biologia Experimental e Tecnológica, Apartado 12, 2781-901 Oeiras, Portugal; 3 Instituto de Tecnologia Química e Biológica António Xavier, Universidade Nova de Lisboa, Av. da República, 2780-157 Oeiras, Portugal

**Keywords:** green chemistry, sustainability, antioxidant activity, high value-added ingredients, bio-residues, *Crocus sativus* L., microwave-assisted extraction, valorization, food by-products, polyphenols

## Abstract

The saffron flower stigmas are used for the saffron spice production while the remaining saffron floral by-products, that are a valuable source of natural bioactive compounds, remain underutilized. The aim of this study was to evaluate the microwave-assisted extraction (MAE) through response surface methodology to obtain high value-added compounds from saffron tepals as ingredients with potential application in the food, pharmaceutical and/or cosmetic industries. A central composite design was applied to optimize process variables: temperature, time and ethanol solvent concentration. Extracts were characterized in terms of total phenolic and total flavonoid content, and antioxidant capacity (ORAC and HOSC assays), being the maximum values obtained: 126.20 ± 2.99 mg GAE/g dry matter; 8.05 ± 0.11 mg CE/g dry matter; 6219 ± 246 μmol TEAC/dry matter; 3131 ± 205 μmol TEAC/dry matter, respectively. Results indicated that the optimal extraction conditions were the combination of low temperature (25 °C)—high extraction time (5 min) using ethanol as solvent (100%). MAE revealed to be an efficient technique to isolate bioactive compounds from saffron floral by-products with a low energy footprint.

## 1. Introduction

Saffron (*Crocus sativus* L.) is used as a spice due to its coloring and flavoring properties, being a traditional Mediterranean plant widely cultivated in different areas, such as Iran, Spain, Greece, Italy, India, Morocco, Algeria and Turkey, among others. The flower of *Crocus sativus* L. is composed of six violet tepals, three golden-yellow stamens, and a white filiform style which culminates in a red stigma divided into three filaments [1].

Saffron is one of the most expensive spices in the world due to its high production costs as the saffron spice stigmas have to be detached manually from the rest of the flower. Around 165.000–230.000 flowers are necessary to produce 1 kg of saffron [2].

In addition to its applications in food, saffron has been traditionally used for health care due to its therapeutic and pharmacological properties [3]. Bioactive compounds such as carotenoids, terpenes, and flavonoids are the major compounds reported in saffron varieties [4]. Despite the well-known saffron characterization, recent studies showed that saffron floral by-products, such as tepals, are rich in valuable bioactive molecules such as flavonols, flavonoid glycosides and anthocyanins, which have positive biological effects such as their antioxidant and antimicrobial activities [5,6]. The valorization of this by-product through the development of innovative high added-value functional ingredients with new applications in food, pharmaceutical and or cosmetic industries could lead to the minimization of the environmental impact and to the potentially increase of saffron demand on the market [7,8].

The extraction of bioactive compounds from saffron floral by-products has been carried out using different conventional extraction methods such as maceration, distillation, and Soxhlet extraction [9]. The need to avoid toxic solvents using environmentally friendly technologies has motivated the development of new processing methods that can be transferred into industrial scale.

In this field, new green energy-saving technologies such as the microwave-assisted extraction (MAE), present several advantages compared to conventional technologies: increase of the extraction kinetics, a shorter extraction time and rapid temperature increase, a higher efficiency and extraction yield as well as a lower energy consumption and cost [10,11,12].

MAE consist on the combination of microwave heating with traditional solid-liquid extraction. This technique is based on the mechanisms of energy transfer as dipole rotation and ionic conduction. The radiation causes the rupture of cells which allow the solvent penetration through the plant matrix. On the other hand, plant material flow outside the cells into the solution [9]. Some of the major factors that could affect the MAE process are the solvent type, temperature, extraction time, and power. Thus, MAE is considered a green technology to save costs, time and energy.

The main focus of this research was to evaluate MAE process efficiency to obtain high added-value antioxidant compounds from saffron floral by-products at laboratory scale. A Central Composite Orthogonal design was used to optimize MAE process conditions (extraction time, temperature, and solvent ratio) for the isolation of bioactive compounds and to study the antioxidant potential of saffron floral by-products. Extracts were characterized in terms of total phenolic (TPC) and flavonoids content (TFC) and antioxidant capacity, evaluated by oxygen radical absorbance capacity and hydroxyl radical scavenging capacity. Therefore, the valorization of this biomass that is currently unexploited could lead to its use as a source of added-value extracts for the potential development of novel bioactive ingredients.

## 2. Materials and Methods

### 2.1. Plant Material

Saffron flowers were obtained during the 2020 harvest season from Castilla La-Mancha region (Spain), cultivated following the requirements established by the Protected Designation of Origin “La Mancha Saffron” and supplied by the Spanish company Verdú Cantó Saffron Spain, S.L. [13]. The flowers were composed of tepals, stamens and styles. Stigmas were detached manually by hand following traditional procedures [13] and the remaining fresh floral by-products were frozen in liquid nitrogen and kept at −80 °C until freeze-dried.

Freeze-drying step took 48 h to constant weight (initial temperature −25 ± 2 °C and pressure 0.220 mbar) in a Christ Alpha 2–4 (B. Braun Biotech International, Melsungen; Germany). The freeze-dried flowers were crushed and sieved through a 500 μm mesh size and kept at −20 °C in polyethylene bags until further analysis.

### 2.2. Experimental Design

Response Surface Methodology (RSM), a collection of statistical and mathematical techniques for improvement and optimize processes, was used to optimize the extraction process and predict the responses which are affected by experimental variables. Using MODDE^®^ software version 12.1 (Sartorius Stedim Biotech, Göttingen, Germany), a Central Composite Orthogonal design (CCO) with three independent variables was applied to evaluate the effect of time (X_1_), temperature (X_2_) and ethanol concentration (X_3_) on the extraction yield, bioactive content and antioxidant activity of saffron flowers. The coded variable levels are summarized in Table 1.

### 2.3. Extraction Procedure

MAE was conducted using Discover SP-CEM MW system (CEM Co., Charlotte, NC, USA), operating at 500 PSI with a maximum output power of 300 W. The variable parameters included radiation time (0.5−5 min), temperature (25−100 °C) and ethanol solvent concentration (0–100%). The mass: solvent ratio (1:10, *w*/*v*) was fixed and the assay was performed with 2 g of sample dissolved in 20 mL of solvent. In this case, 14 extractions were carried out, following the designed conditions indicated in Table 1. Once the extraction was completed, the extracts were dried by a CentriVap^®^ Concetrator (Labconco, Kansas City, MO, USA) at 37 °C during 24 h.

The extraction yield was determined using Equation (1). The characterization experiments (bioactive composition and antioxidant capacity) were conducted in triplicate. For these assays dry extracts were reconstituted in 50% ethanol (*w*/*v*).

Yield% = ((weight of extract dried)/(initial weight of extract)) × 100(1)

### 2.4. Extract Characterization

#### 2.4.1. Total Phenolic Content

TPC was determined using the Folin Ciocalteau colorimetric method [14]. Briefly, 10 μL of the saffron floral extracts were mixed with 230 μL of milli-Q water, 15 μL of Folin-Ciocalteu’s reagent and 45 μL of sodium carbonate solution (35%). The samples were stirred and incubated at room temperature during 1 h under dark conditions. Gallic acid (1000 mg/L) was used as a reference standard (7.5–240 mg/L) and absorbance was measured at 765 nm in a microplate reader (SynergyHT, Biotek, Winooski, VT, USA). The results were expressed as mg gallic acid equivalent (mg GAE) per gram of dry matter.

#### 2.4.2. Total Flavonoid Content

TFC was determined as described by Çam and Hışıl [15]. Briefly, 1 mL of sample were mixed with 4 mL of water and 300 µL of sodium nitrite (5%) solution. After 5 min, 300 µL of aluminum trichloride (10%) solution were added. After more 6 min, 2 mL of sodium hydroxide (1M) solution were added, and the volume was adjusted to 10 mL with water. Catechin (1000 mg/L) was used for quantification (20–100 mg/L) and absorbance was measured at 510 nm in a spectrophotometer (UV/Vis Spectrophotometer T80; PG Instruments Limited, Lutterworth, UK). The results were expressed as mg of catechin equivalents (mg CE) per gram of dry matter.

#### 2.4.3. Oxygen Radical Absorbance Capacity Assay

The ORAC assay method measures the capacity of the antioxidant molecules, present in the extracts, to protect the disodium fluorescein (FL) from oxidation by peroxyl radicals. The assay was carried out following the method described by Serra et al. [16]. This assay measured the ability to inhibit the oxidation of fluorescein (3 × 10^−4^ mM) catalyzed by peroxyl radicals generated from AAPH (2,2-Azobis (2-methylpropionamidine) dihydrochloride) using a microplate fluorescent reader (FL800 Bio-Tek Instruments, Winooski, VT, USA). Trolox (1000 µM) was used as a reference standard (5–30 µM) and the results were expressed as micromoles of trolox equivalents antioxidant capacity (μmol TEAC) per gram of dry matter.

#### 2.4.4. Hydroxyl Radical Scavenging Capacity Assay

HOSC method was used to evaluate the hydroxyl radical scavenging capacity of samples. The assay was performed using the method described in Moore et al. [17]. Briefly, in a microplate fluorescent reader, fluorescein (9.96 × 10^−8^ M) was used as a probe and the reaction of ferric chloride (3.42 mM) and hydrogen peroxide (0.20 M) as a source of hydroxyl radicals. Trolox (1000 µM) was used as a reference standard (5–30 µM) and the results were expressed as micromoles of trolox equivalents antioxidant capacity (μmol TEAC) per gram of dry matter.

### 2.5. Statistical Analysis

Results were expressed as the mean ± standard deviation. The mean comparisons were carried out using an analysis of variance (ANOVA) and the Tukey multiple range test, using GraphPad Prism 5 software (GraphPad Software, Inc., La Jolla, San Diego, CA, USA). The significant differences were stablished as (*p* < 0.05). All determinations were carried out in triplicate.

## 3. Results and Discussion

### 3.1. Bioactive Content

A design of experiments (DOE) was planned to obtain high added value compounds from saffron floral by-products. To optimize the extraction procedure and evaluate MAE as a green extraction method, the effect of process variables on the mass yield, TPC and TFC, and the antioxidant capacity (ORAC and HOSC) were evaluated on a CCO design with three independent variables, time (X_1_), temperature (X_2_) and ethanol concentration (X_3_).

**Figure 1:**
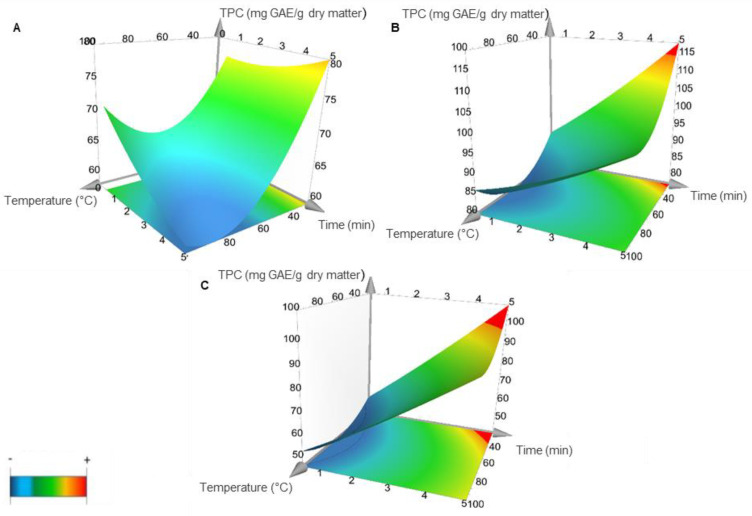
Response surface plot representing the effects of time, temperature and solvent ratio on Total Phenolic Content (TPC) from saffron floral by-products. (**A**) Ethanol concentration was kept constant at 0%. (**B**) Ethanol concentration was kept constant at 50%. (**C**) Ethanol concentration was kept constant at 100%. Lower values are represented in blue and higher values in red.

The results of the interaction effects of the three independent variables studied in MAE on phenolics compounds extraction are shown in Figure 1. As it can be seen in the response surface plot, the interaction between temperature and time, keeping constant the solvent ratio (ethanol concentration), can be exploited to increase the affinity and specificity of the extraction. By changing the conditions, we can adjust the solvent polarity for the desired extraction. Nevertheless, the values were lower using a high temperature and time (blue surfaces in the Figure 1A). This fact might be due either to an initial thermal degradation of free phenolic compounds in the floral extracts at 5 min, since other studies have showed a significant decrease in TPC values during the thermal treatment on grape marc at 80 °C [18], and to the decrease in the dielectric constant and polarity of water and ethanol solvents at high temperatures with a reduction in their capability to dissolve polar compounds [19]. TPC indicated that increasing the extraction time while decreasing the temperature and keeping constant the ethanol ratio at 50 or 100% (Figure 1B,C, respectively), lead to an increase in the extraction yield of these bioactive compounds (red surfaces).

The empirical values of the TPC obtained for the MAE of bioactive compounds are showed in Table 2. The values of TPC obtained by MAE from Spanish saffron flowers (maximum value: 126.20 ± 2.99 mg GAE/g dry matter) were higher than those reported in previous research using saffron floral bio-residues from Italy, where values of 4000 mg GAE/100 g of dry matter were reported [20] when MAE was used as extraction method, and a TPC richness of 40–50 mg GAE/g of dry extract, using MAE as a pretreatment to a conventional solid–liquid extraction reported by Álvarez et al. [12].

**Figure 2:**
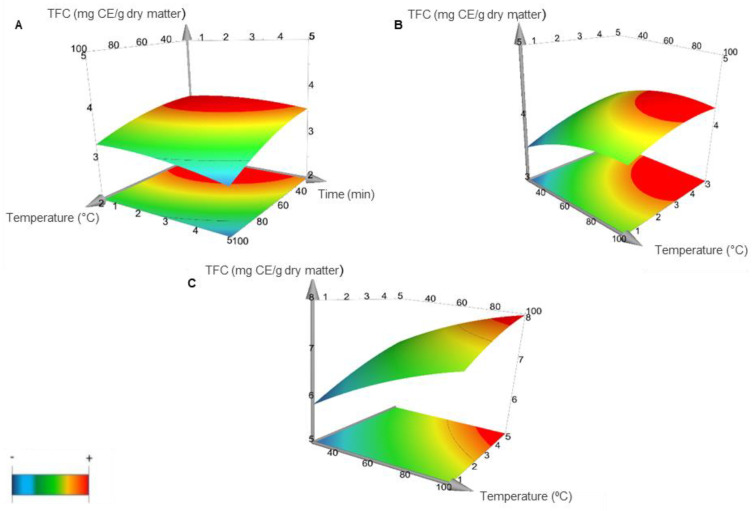
Response surface plot representing the effects of time, temperature and solvent ratio on Total Flavonoids Content (TFC) from saffron floral by-products. (**A**) Ethanol concentration was kept constant at 0%. (**B**) Ethanol concentration was kept constant at 50%. (**C**) Ethanol concentration was kept constant at 100%. Lower values are represented in blue and higher values in red.

With respect to TFC, the results of the interaction effects of the three independent variables studied in MAE from saffron floral by-products are shown in Figure 2. It illustrates the interaction between temperature and time, keeping constant solvent ratio during the extraction process. The regression analysis of data showed that, for a 95% confidence level (*p* < 0.05), flavonoids concentration in the extract was positively affected by temperature and solvent ratio. As it can be seen in the response surface plot, the shape of the Figure 2C indicated that, keeping constant ethanol ratio at a level of 100%, flavonoids extraction was enhanced (red surface) with an increase in the extraction temperature.

The empirical values of TFC obtained for the MAE of bioactive compounds are shown in Table 2. The results of TFC were more similar between the different extraction experiments than those of TPC. However, the highest concentrations of flavonoids were found in the extractions 5, 6, 7 and 8, being the values between 5.62–8.05 mg CE/g dry matter where the concentration of ethanol used was the 100%. These results are in accordance with previous research in the literature by other authors that had also studied the variables of temperature, time and solvent ratio for MAE to extract anthocyanins from Iranian saffron tepals. The results indicated that the solvent ratio was the factor which had the most significant effect in the extraction of bioactives [21]. Furthermore, all experiments were conducted at a maximum irradiation power (300 W), however, its effects on flavonoids extraction should also be studied. Previous research evaluated the effect of the power in MAE and has suggested that the microwave power has a significant effect on extracting bioactive compounds from saffron petals because of the fact that the irradiation of microwave may accelerate the rupture of plant cells due to a rapid increase of pressure and temperature [22].

The values of TFC obtained by MAE were in accordance with the ones described by Sun et al. [23], reporting values lower than 10 mg/g for TFC in saffron tepals methanol extracts obtained with ultrasound agitation (150 W, 40 °C, 40 min). Thus, MAE was an efficient technique to extract saffron bioactives in shorter times compared to other processes.

Additionally, according to Table 2 and to the RSM results, the extraction yield increased by the increment of the extraction temperature (62.5 °C and 100 °C), being the experiment 9 (62.5 °C and 50% ethanol), the one with the optimum conditions leading to a 36.75% of yield. The regression analysis of data showed that, for a 90% confidence level (*p* < 0.10), extraction yield was only significantly affected by extraction temperature. The extraction yields obtained were higher than those reported by Hashemi Gahruie et al. [22] in which the yield values using MAE to extract bioactive compounds from Iranian saffron tepals were between 8.07 and 19.42%.

Therefore, according to the results shown in Table 2, the parameters used in the experiment 6 in MAE (25 °C, 5 min, 100% ethanol) were the optimum for increasing the extraction yield of both TPC and TFC. The maximum concentrations obtained for TPC and TFC was 126.20 ± 2.99 mg GAE/g dry matter and 6.80 ± 0.33 mg CE/g dry matter, respectively. The regression analysis of data showed that, for a 90% confidence level (*p* < 0.10), phenolic concentration in the saffron floral by-product extract was positively influenced by extraction time and ethanol concentration. However, the effect of temperature on the phenolic concentration was not significant while it had a significantly positive effect on mass yield.

The analysis of the model indicated that the optimal extraction conditions, for these bioactive compounds, were the combination of low temperature (25 °C) using only ethanol as solvent (100%) with longer extraction times (5 min). However, looking in more detail into the TPC extraction, it can be observed that to obtain a high concentration of phenolic compounds, the conditions varied according to the extraction temperature chosen, so in order to reach values higher than 80 mg GAE/g dry matter from saffron flowers, combinations with higher temperatures would be suitable, given to the fact that high temperatures will increase the extraction yield. TPC was enhanced with half concentration of ethanol at high temperatures, that could be explained by the increase of the pressure of the solvent system, which led to the breakdown of the cell walls of the plant material, improving the penetration of solvent across the sample matrix and increasing the release of the bioactive compounds. Since the boiling point of ethanol is lower than water (78 °C), this increase in pressure only occur when using ethanol as primary solvent (50 or 100%) [24].

Saffron floral by-products revealed to be a high-level source of phenolics and flavonoids compounds, efficiently isolated by MAE, a greener extraction method than the commonly used organic solvents. These saffron floral bio-residues could be used as natural sources of bioactive compounds with different biological activities, that can be used in the development of innovative functional foods, nutraceuticals or in cosmetic applications.

### 3.2. Antioxidant Activity

A number of methods have been developed to measure the efficiency of dietary antioxidants either as pure compounds or in food extracts. These methods focus on different mechanisms of the antioxidant defense system, i.e., scavenging of oxygen and hydroxyl radicals, reduction of lipid peroxyl radicals, inhibition of lipid peroxidation, or chelation of metal ions. One of the in vitro methods commonly used to measure the antioxidant capacity of food constituents is ORAC assay, since it determines the potential to scavenge harmful oxygen reactive species that are biologically relevant radicals, such as peroxyl radicals, that are involved in the lipid oxidation in food systems [25]. Other in vitro method to determine the antioxidant capacity is HOSC assay that measures the ability of the extracts to scavenge hydroxyl radicals generated by hydrogen peroxide. In this work, the two methods were used to evaluate the antioxidant capacity of the saffron floral extracts obtained by MAE.

The empirical results for ORAC and HOSC assays are shown in Table 2. The regression analysis of data showed that, for a 95% confidence level (*p* < 0.05), the antioxidant activity evaluated by ORAC was significantly affected by solvent ratio, namely ethanol concentration in the extraction process. Moreover, antioxidant activity evaluated by HOSC was significantly affected by extraction time and temperature. The use of two methods for screening the antioxidant activity of the extracts can reveal that various distinct compounds are extracted under different conditions. According to the results, the parameters used in the experiment 6 for MAE were the optimum regarding both, ORAC and HOSC assays, reaching values of 5128 ± 303 μmol TEAC/g dry matter and 3131 ± 205 μmol TEAC/g dry matter, respectively. In all these experiments, the temperature was below 100 °C, and the ethanol solvent ratio was always above 50%.

The reactions involved in those analytical methods are associated to enzymatic and non-enzymatic oxidation reactions, these results can be explained by the oxidation of phenolic compounds at high temperatures that lead to a loss in their antioxidant activity [26]. This information was in accordance with previous studies of natural compounds that have investigated the effect of drying temperatures in the antioxidant activity of grape pomace peels extracts. The authors have found that the antioxidant activity at 120 °C was lower (1.7 times) than that obtained at 20 °C [27,28].

ORAC results for the floral extracts were comparable with those reported by Sun et al. [23] in which saffron tepals showed a strong oxygen radical scavenging ability, even higher than that of the stamen extracts, but for HOSC assay, any study has been published to date for saffron floral samples.

Many natural substances such as carotenoids, tocopherols, and polyphenols can act as antioxidants and are widely spread within food and plants. Flavonoids and other polyphenols can scavenge free radicals thus delaying lipid autoxidation. Several studies have been carried out to correlate polyphenolic composition with its antioxidant properties of natural extracts.

In this work, the antioxidant activity showed correlation with TPC values, since in the experiments 6 and 9, saffron flowers extracts presented a high concentration of phenolic compounds and a high antioxidant ability. Regarding TFC values, the results showed that the optimal experiment was also number 6, using 100% of ethanol as extraction solvent during 5 min at low temperature (25 °C).

**Figure 3:**
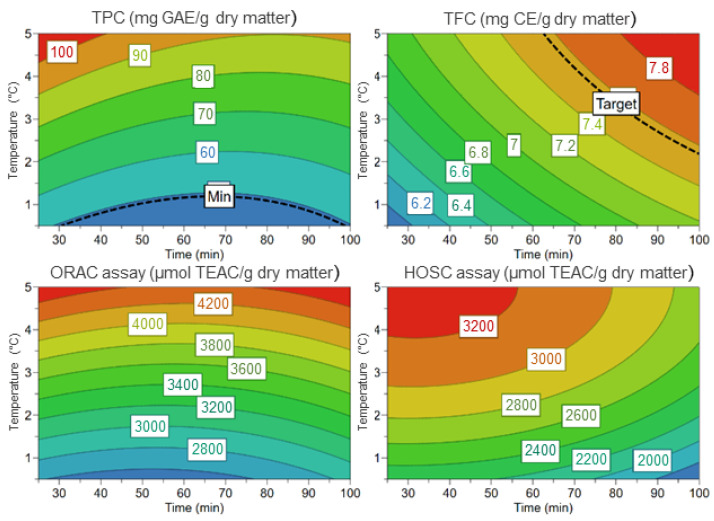
Response contour plots representing the effects of time and temperature at a constant ethanol concentration (100%), on all extraction responses. Lower values are represented in blue and higher values in red.

The best extraction results and the interaction effects of the variables studied for TPC, TFC and antioxidant activity are illustrated in Figure 3. As it can be seen in the response contour plot, at low temperatures (25 or 62.50 °C), TPC and antioxidant capacities by ORAC and HOSC assays were higher (red surface) than those observed at 100 °C.

These findings were comparable to previous research by Sólyom et al. [18] that reported that the antioxidant activity and the total polyphenol content on grape marc decreased after a treatment at high temperature, that may be due to a degradation of the bioactive compounds. Gallo et al. [29] also applied MAE for the recovery of saffron phenolic compounds. Using ethanol (50%) at 200 W and low temperatures (50 °C) during 18 min obtaining extracts rich in total phenolic content with high antioxidant activity. Their results also suggested that extracts obtained by MAE showed a higher antioxidant ability (45 times) than the one obtained by ultrasound assisted extraction. MAE was a better technique to isolate interesting bioactive compounds from *Crocus sativus* L., most likely because of the good interaction between the solvent and the plant matrix during MAE.

The ORAC and HOSC results revealed the antioxidant activities of saffron floral by-products. This antioxidant power would be due to their bioactive content, such as polyphenols, since several research have reported that spices and herbs, with high phenolic content, are excellent sources of natural antioxidant compounds [30]. Nevertheless, the relationship between the structure of polyphenols and their antioxidant power is not yet well known [28]. Therefore, saffron floral by-products could be a source of natural antioxidants resulting from the saffron spice handling and processing and can be used to develop nutraceutical or functional food products with positive effects in human health such as the protection against oxidative stress [31].

## 4. Conclusions

This study explored the effects of different processing parameters such as time, temperature and ethanol concentration in MAE on saffron floral by-products, in terms TPC, TFC, and antioxidant activity (ORAC and HOSC assays). At laboratory scale, the results showed that the optimal MAE conditions for the extraction bioactive compounds were using ethanol as primary solvent (50 or 100%) and low temperatures. Therefore, this information could help to choose the most appropriate extraction method and parameters to obtain compounds of interest from natural plant sources, including scale-up to industrial level.

From these findings, it can be concluded that MAE was an efficient technique that allows to obtain high added value compounds from saffron floral by-products with low energy footprint. Additionally, this research provides new information about the functional compounds present in saffron floral by-products extracts, representing an important source of natural antioxidant compounds and that could be considered as a source of promising bioactive ingredients for the development of functional foods and for other human health applications.

## Figures and Tables

**Table 1:** The CCO matrix of MAE of the experimental design.

	Independent Variables: MAE Conditions		
Experiments	Time (min) (X_1_)	Temperature (°C) (X_2_)	Ethanol Concentration (%) (X_3_)
1	0.5 (−1)	25 (−1)	0 (−1)
2	5 (+1)	25 (−1)	0 (−1)
3	0.5 (−1)	100 (+1)	0 (−1)
4	5 (+1)	100 (+1)	0 (−1)
5	0.5 (−1)	25 (−1)	100 (+1)
6	5 (+1)	25 (−1)	100 (+1)
7	0.5 (−1)	100 (+1)	100 (+1)
8	5 (+1)	100 (+1)	100 (+1)
9	6.2 (+1.35)	62.5 (0)	50 (0)
10	3.15 (0)	11.75 (−1.35)	50 (0)
11	3.15 (0)	113.24 (+1.35)	50 (0)
12	3.15 (0)	62.5 (0)	50 (0)
13	3.15 (0)	62.5 (0)	50 (0)
14	3.15 (0)	62.5 (0)	50 (0)

**Table 2:** Empirical results of total phenolic content (TPC), total flavonoid content (TFC), yield, and ORAC and HOSC assays for the microwave-assisted extraction of bioactive compounds from saffron floral-by products ^1^.

Extraction	Time (min)	Temperature (°C)	Ethanol (%)	TPC (mg GAE/g Dry Matter)	TFC (mg CE/g Dry Matter)	Yield (%)	ORAC (μmol TEAC/g Dry Matter)	HOSC (μmol TEAC/g Dry Matter)
1	0.50	25.00	0	93.87 ± 3.33 cd	3.56 ± 0.23 efg	20.65	4777 ± 352 bcd	2286 ± 583 abc
2	5.00	25.00	0	80.54 ± 1.56 ef	3.33 ± 0.039 fg	25.15	2679 ± 504 fg	2034 ± 253 cd
3	0.50	100.00	0	54.82 ± 4.33 gh	3.26 ± 0.25 fg	36.40	2170 ± 304 g	1281 ± 230 d
4	5.00	100.00	0	58.62 ± 4.44 g	3.13 ± 0.03 g	32.35	2175 ± 268 g	2117 ± 127 bcd
5	0.50	25.00	100	52.12 ± 1.75 gh	5.62 ± 0.02 c	23.65	2019 ± 439 g	1716 ± 44 cd
6	5.00	25.00	100	126.20 ± 2.99 a	6.80 ± 0.33 b	17.10	5128 ± 303 bc	3131 ± 205 a
7	0.50	100.00	100	49.19 ± 1.67 h	7.41 ± 0.35 ab	29.70	3451 ± 443 ef	1623 ± 277 cd
8	5.00	100.00	100	75.47 ± 1.02 f	8.05 ± 0.11 a	27.00	4026 ± 84 de	2124 ± 383 bcd
9	6.20	62.50	50	105.50 ± 3.05 b	4.23 ± 0.14 de	36.75	5027 ± 351 bc	2444 ± 112 abc
10	3.15	11.75	50	85.13 ± 2.13 e	4.15 ± 0.42 de	31.80	5641 ± 384 ab	2407 ± 315 abc
11	3.15	113.24	50	120.70 ± 3.69 a	3.67 ± 0.15 defg	27.75	4556 ± 390 cd	1779 ± 272 cd
12	3.15	62.50	50	86.97 ± 1.38 de	4.34 ± 0.38 d	23.60	4637 ± 37 cd	1995 ± 131 cd
13	3.15	62.50	50	96.77 ± 3.46 bc	3.99 ± 0.22 def	34.15	3278 ± 121 ef	2240 ± 265 bc
14	3.15	62.50	50	79.28 ± 3.14 ef	4.14 ± 0.31 de	25.70	6219 ± 246 a	2944 ± 407 ab

^1^ Means ± standard deviation in the same column followed by different lowercase letters indicate statistically significant differences at (*p* ≤ 0.05) for each extraction (*n* = 3).
